# CHD5 is down-regulated through promoter hypermethylation in gastric cancer

**DOI:** 10.1186/1423-0127-16-95

**Published:** 2009-10-19

**Authors:** Xian Wang, Kenneth KK Lau, Leo KY So, Yun Wah Lam

**Affiliations:** 1 Department of Biology and Chemistry, City University of Hong Kong, Hong Kong SAR, China; 2 Department of Medical Oncology, Biomedical Research Center, Sir Runrun Shaw Hospital, Zhejiang University, Hangzhou, China; 3 Department of Microbiology, The University of Hong Kong, Hong Kong SAR, China

## Abstract

**Background:**

Nonhistone chromosomal proteins in concert with histones play important roles in the replication and repair of DNA and in the regulation of gene expression. The deregulation of these proteins can contribute to the development of a variety of diseases such as cancer. As a nonhistone chromosomal protein, chromodomain helicase DNA binding protein 5 (CHD5) has recently been identified as the product of a novel tumor suppressor gene (TSG), promoting the transcription of p19^*ink4a *^and p16^*arf*^. The inactivation of CHD5 was achieved partly through genetic deletion since it is located in 1p36, a region frequently deleted in human tumors. In this study, we aim to study the involvement of CHD5 in gastric cancer, the second most common cancer worldwide.

**Methods:**

CHD5 expression in a panel of gastric cancer cells were determined by quantitative RT-PCR. The methylation of CHD5 was evaluated by methylation specific PCR and bisulfite genome sequencing. The effect of CHD5 on growth of gastric cancer cells was tested by colony formation assay.

**Results:**

CHD5 expression was down-regulated in all of gastric cancer cell lines used (100%, 7/7) and significantly restored after pharmacological demethylation. Methylation of CHD5 promoter was detected in all of seven gastric cancer cell lines and in the majority of primary gastric carcinoma tissues examined (73%, 11/15). Finally, ectopic expression of CHD5 in gastric cancer cells led to a significant growth inhibition.

**Conclusion:**

CHD5 was a TSG epigenetically down-regulated in gastric cancer.

## Background

All eukaryotic organisms have developed elaborate ways of packaging DNA into chromatin through the dynamic interactions of various DNA-associated proteins. Such packaging is not only important for the storage of genetic information with high fidelity and integrity, but also the transfer of genetic information from DNA to RNA in a tightly controlled manner. Proteins that bind to DNA to form chromatin are traditionally divided into two general classes: histones and nonhistone chromosomal proteins. Histones are a group of highly conserved DNA binding proteins and their various post-translational modifications constitute the 'histone code' that guides the packaging of DNA or chromatin remodeling. The histone code is initiated, maintained and interpreted largely by nonhistone chromosomal proteins [1-4]. For example, the acetylation of lysine residues on histone tails by histone acetyltransferases (HATs) neutralizes their charge and decreases the affinity of histones with DNA, making DNA accessible for transcriptional factors to initiate gene transcription. Conversely, the deacetylation of these residues by histone deacetylases (HDACs) restores this affinity and can withdraw DNA from transcriptional machinery [5]. In addition to acetylation, phosphorylation and methylation of histone tails are important for the dynamic association of DNA with transcriptional machinery and other chromosomal proteins [6-8]. Nonhistone chromosomal proteins play important roles in the interpretation of histone code by forming chromatin remodeling complexes. Both histones and nonhistone chromosomal proteins are important for the regulation of gene expression, DNA replication and DNA repair. The deregulations in the expression and activity of these proteins could result in the development of a variety of diseases such as cancer [9-13].

In a recent study, chromodomain helicase DNA binding protein 5 (CHD5) was identified as a novel tumor suppressor gene (TSG) in neuroblastoma [14]. CHD5 belongs to a superfamily of SWI2/SNF2-related ATPases, one major group of nonhistone chromosomal proteins. CHD5 encodes a unique combination of functional domains consisting of two N-terminal chromodomains, followed by a SWI2/SNF2-like ATPase/helicase domain and a DNA-binding domain [14]. By regulating chromatin structure, CHD5 can promote the expression of p19^arf ^that functions to stabilize p53, the tumor suppressor inactivated in more than half of human cancers [15]. CHD5 is present at a gene locus (1p36.31) deleted in about 35% of neuroblastoma [16]. CHD5 was previously thought to be specifically expressed in the nervous system, but its role in cancer in other tissues is starting to emerge [17]. CHD5 gene was found significantly deleted in glioma [18]. Apart from gene deletion, CHD5 can be suppressed by other mechanisms. In some cases of neuroblastoma, there are evidence that CHD5 expression is epigenetically suppressed by promoter hypermethylation [19], although this observation was not confirmed by another study [20]. Recently, the CHD5 promoter has been found to be methylated in small subsets of breast (4.4%), colon (10%), ovarian (15%) and glioma (17%) tumors [17,20], suggesting epigenetic silencing of CHD5 by methylation may play a partial role in tumorigenesis in these tissues. Here we found that, in contrast to other types of cancer reported so far, CHD5 was frequently hypermethylated in gastric cancer (73% of tumors and 100% cell lines). The ectopic expression of CHD5 in gastric cancer cells led to a significant growth inhibition. This striking correlation of the epigenetic suppression of CHD5 and gastric cancer suggests a previously unknown relationship between this TSG and gastric tumorigenesis.

## Methods

### Tissue culture and RNA/DNA extraction

All gastric cancer cell lines (AGS, Kato III, MKN28, MKN45, SNU1, SNU16 and NCI-N87) were obtained from Riken Gene Bank (Tsukuba, Ibaraki, Japan) and American Type Culture Collection (ATCC, Manassas, VA, USA). All cancer cell lines were established from carcinomas of gastric epithelial cells. Unless specifically indicated, cells were cultured in RPMI 1640 medium (Invitrogen, Carlsbad, CA, USA) supplemented with 10% fetal bovine serum at 37°C with 5% CO_2_, and 95% humidity. For pharmacological demethylation, cells were treated with 5 μM 5-aza-2'-deoxycytidine (Aza) (Sigma, St Louis, MO, USA) for three consecutive days [21]. Aza was replenished every 24 hours. An equivalent concentration of the vehicle (DMSO) was used as the control. For the primary tissues, the normal gastric tissues were defined as non-inflammation and non-tumor tissues. All gastric carcinoma tissues are adenocarcinoma tissues. Total RNA and genomic DNA was extracted using Trizol reagent (Invitrogen) according to the manufacturer's instruction.

### Quantitative real time RT-PCR

Reverse transcription reaction was performed using 1 μg of total RNA with Reverse Transcription System (Promega, Madison, WI, USA). The mRNA expression levels of the CHD5 were determined by quantitative real-time RT-PCR SYBR Green Master Mix Kit (Applied Biosystems, Foster City, CA, USA). Glyceraldehyde-3-phosohate dehydrogenase (GAPDH) was used as an internal control of RNA integrity. Primers used for CHD5 RT-PCR were CHD5-F: 5'-AGTTCCGTGTGAGGATGAAC and CHD5-R: 5'-TCAAGGCTGACGTGTTCAAG.

### Methylation specific PCR (MSP)

Methylation status of CHD5 was determined by MSP using bisulphate modified genomic DNA as the template. Genomic DNA was bisulfite-treated with Zymo DNA Modification Kit (Zymo Research, Orange, CA, USA) according to the protocol provided. MSP was carried out for 40 cycles with annealing temperature at 62°C, as previously described [22]. Methylation-specific primers were: CHD5M-F: 5'-GTTCGGGGTTTAGCGTTTTC (from -525 to -506 relative to the transcription start site) and CHD5M-R: 5'-GAAACTTAACGAACCCGAACG (from -438 to -418), and unmethylation-specific primers were: CHD5U-F: 5'-GGGTTTGGGGTTTAGTGTTTTT and CHD5U-R: 5'-TCAAAACTTAACAAACCCAAACA. All primers were confirmed previously for not amplifying any unbisulfited DNA.

### Bisulfite genome sequencing (BGS)

Bisulfite-treated DNA was amplified using BGS primers, CHD5-BF: 5'-GTTGTAAATTAGATTTATAGTTTT (from -724 to -701) and CHD5-BR: 5'-GCAAATTAAAAAACTAATCCTAAA (from -324 to -301). PCR products were purified with Illustra GFX™ PCR and gel band purification kit (GE Healthcare life science, Uppsala, Sweden) and cloned into pCR4-TOPO vector for sequencing (Invitrogen). At least 6 colonies were randomly chosen for plasmid extraction and sequencing analysis.

### Construction of CHD5 expression plasmids

The CHD5 expression plasmid was constructed by cloning of the full-length CHD5 open reading frame into mammalian expression vector pcDNA3.1. The CHD5 open reading frame was amplified from normal stomach cDNA using high fidelity PFU DNA polymerase (Invitrogen) and cloned into pcDNA-TOPO4 (Invitrogen). After sequencing validation, the insert was subcloned into pcDNA3.1 with the restriction enzymes Hind III and Xba I.

### Colony formation assay

Transiently transfected AGS cells with empty pcDNA3.1 or pcDNA3.1-CHD5 were used for monolayer colony formation assay. Cells were cultured overnight in a 12-well plate (1.0 × 10^5^/well) and transfected with pcDNA3.1 or the CHD5-expressing vector using lipofectamine™ 2000 (invitrogen). 48 hours later, the transfectants were re-plated in triplicate and cultured for 10-15 days in complete RPMI1640 medium containing G418 (400 μg/ml). Surviving colonies were stained with Gentian Violet after methanol fixation and colonies exceeding certain size (≥ 50 cells) were counted. The experiments were repeated three times.

## Results

### Down-regulation of CHD5 expression in gastric cancer cell lines

**Figure 1:**
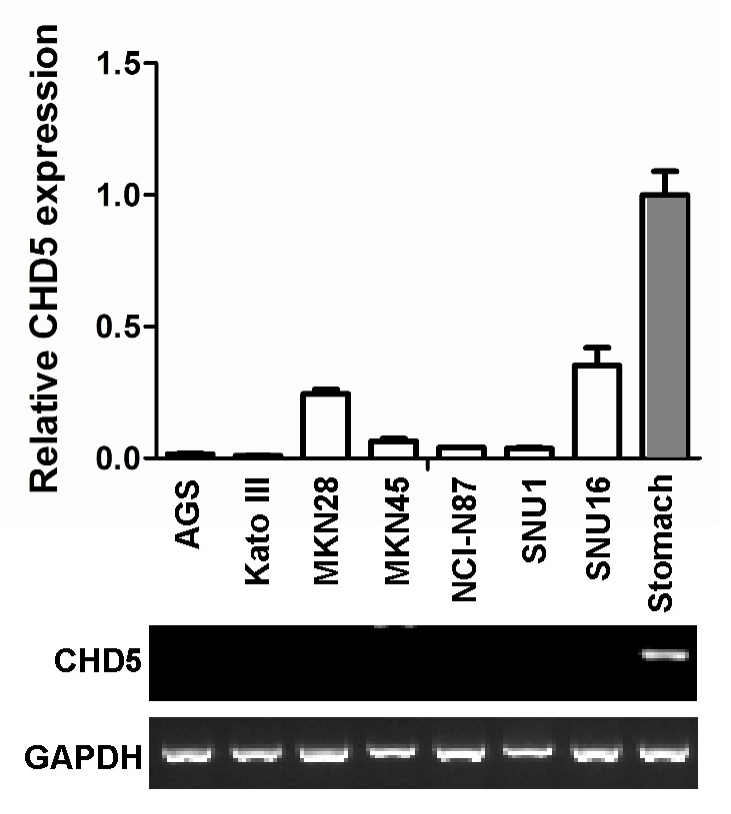
**CHD5 is down-regulated in gastric cancer cell lines**. The expression of CHD5 in gastric cancer cell lines was determined by RT-PCR. GAPDH was used to normalize the CHD5 expression. The normal stomach tissue (stomach) was used as the reference. Results of quantitative real-time RT-PCR was shown in upper panel and the result of conventional RT-PCR was shown in lower panel.

The expression of CHD5 in gastric cancer cell lines was determined by quantitative RT-PCR. While CHD5 was highly expressed in normal gastric tissues, its expressions were down-regulated in all 7 gastric cancer cell lines (AGS, Kato III, MKN28, MKN45 and NCI-N87 SNU1 and SNU16) (Fig. 1).

### Promoter hypermethylation of CHD5 in gastric cancer cell lines and primary gastric carcinoma tissues

**Figure 2:**
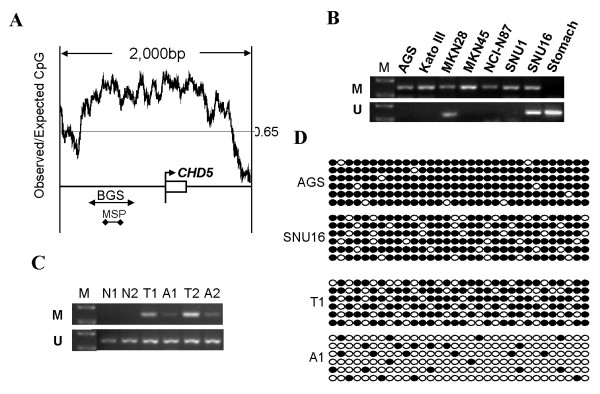
**CHD5 promoter is hypermethylated in gastric cancer**. **A**, CHD5 has a typical CpG island (CGI) around its exon 1. CGI was plotted by GeneTool program. The positions of BGS and MSP primers were indicated as arrows. **B**, The methylation status of CHD5 promoter in gastric cancer cells was determined by methylation specific PCR. M: methylation; U: unmethylation. **C**, The methylation status of CHD5 promoter in primary gastric cancer tissues was determined by methylation specific PCR as in B. Normal gastric tissues and adjacent non-tumor tissues were used as controls. N1 and N2 are normal gastric tissues. T1 and T2 indicate gastric carcinoma tissues while A1 and A2 represent adjacent non-tumor tissues. Represented results were shown. **D**, Methylation of CHD5 promoter in gastric cancer cell lines and primary gastric carcinoma tissues was confirmed by BGS. Each circle indicates one CpG site and circles filled in black represent methylated CpG sites. One row of circles represents a single colony.

A typical CpG Island (CGI) was found around CHD5 exon 1 using the following criteria: GC content >55%, ObsCpG/ExpCpG >0.65, and length >500 bp (Fig. 2A). The methylation status of this CGI in gastric cancer cells was determined by methylation specific PCR (MSP). As shown in Fig. 2B, full or partial methylation was detected in all of 7 gastric cancer cell lines we examined. In consistent with the data on gastric cell lines, the CHD5 promoter was also methylated in the majority of primary gastric carcinoma tissues tested (73%, 11/15) (Fig. 2C). Importantly, methylation of the CHD5 promoter was either undetected or weakly detectable in normal tissues adjacent to the tumors of the same patients and in gastric tissues of normal subjects. Moreover, methylation of CHD5 promoter in gastric cancer cell lines and gastric carcinoma tissues was confirmed by bisulfite genome sequencing (BGS) (Fig. 2D). Taken together, CHD5 was predominately silenced in gastric cancer and promoter hypermethylation appeared to be the major mechanism of CHD5 silencing in tumorigenesis of this tissue.

### Up-regulation of CHD5 expression after Aza treatment

**Figure 3:**
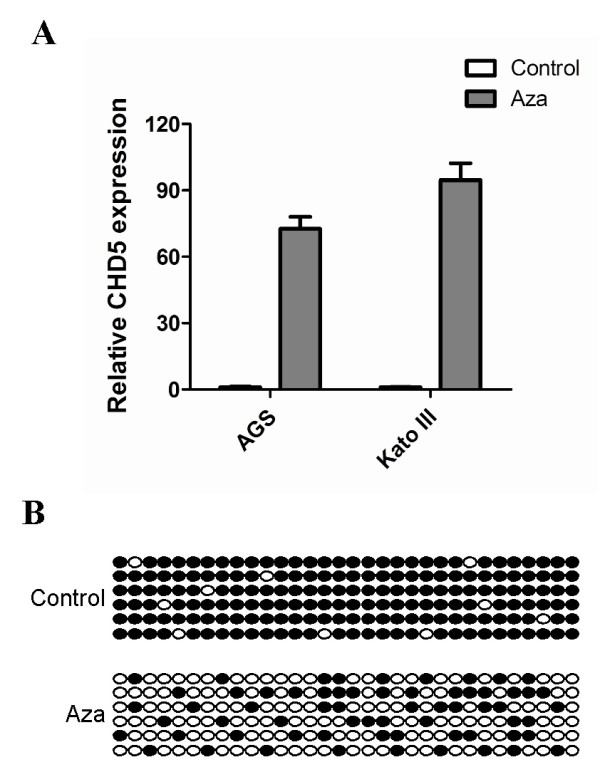
**Pharmacological demethylation reactivates CHD5 expression in gastric cancer cell lines**. **A**, Relative CHD5 expressions before and after Aza treatment were determined by RT-PCR as in Fig. 1. GAPDH was used to normalize the template amount. **B**, Demethylation of CHD5 promoter in AGS cells after Aza treatment was confirmed by BGS as in Fig. 2D.

To further confirm the promoter CGI hypermethylation-mediated CHD5 silencing in gastric cancer cell lines, CHD5 expressions in AGS and Kato III before and after demethylation reagent Aza treatment were analyzed. Both cell lines exhibit complete methylation of the CHD5 promoter. CHD5 expression in these two cell lines were significantly increased after Aza-induced demethylation of CHD5 promoter (Fig. 3A and 3B), demonstrating that CHD5 is indeed epigenetically silenced in gastric cancer.

### Growth inhibitory function of CHD5

**Figure 4:**
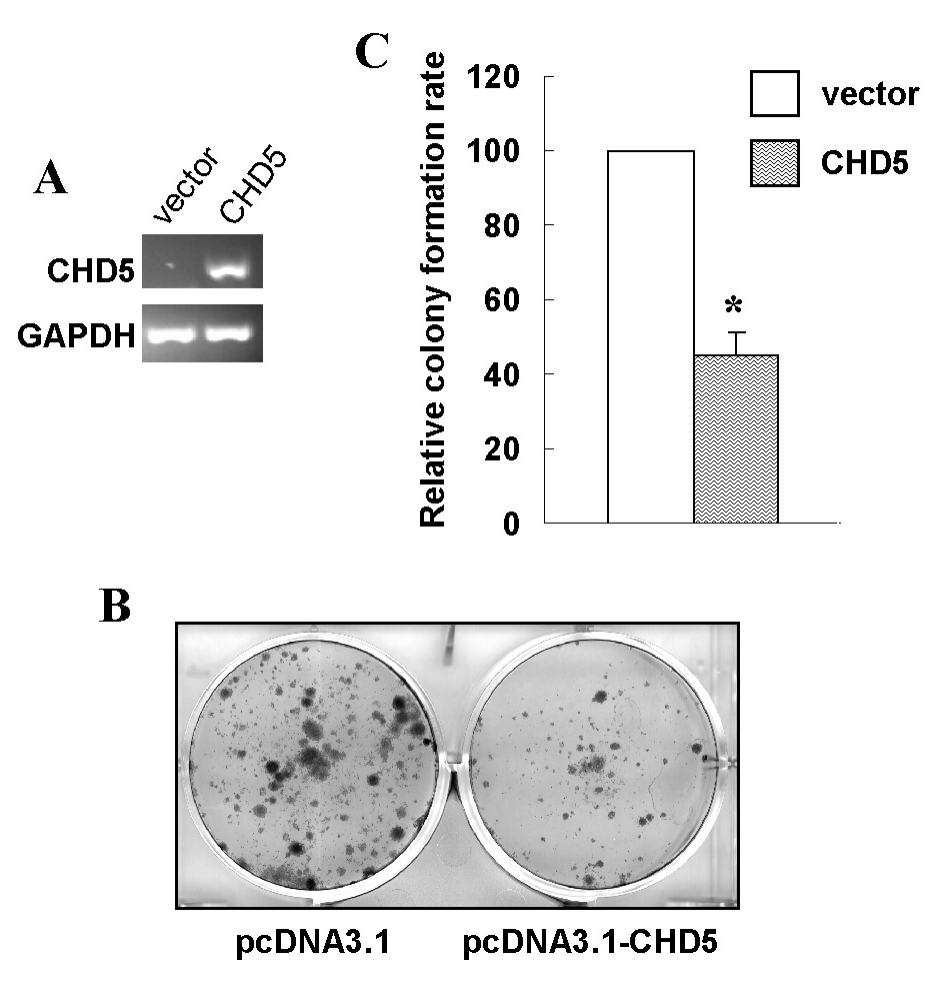
**CHD5 inhibits growth of gastric cancer cell line AGS**. The effect of ectopic CHD5 expression on tumor cell growth was investigated by the monolayer colony formation assay. **A**, CHD5 expression in AGS cells after transfection was determined by RT-PCR. The photograph of colonies formed by AGS cells transfected with pcDNA3.1 (vector) or pcDNA3.1-CHD5 (CHD5) was shown in **B**. **C**, Quantitative analyses of colony numbers are shown as values of mean ± standard deviation. *P *values were calculated using student's t-test. The asterisk indicates statistical significant difference (*p *< 0.01).

The tumor suppression property of CHD5 in gastric cancer cells was investigated by a gain-of-function strategy. Full open reading frame (ORF) of CHD5 was cloned into mammalian expression vector pcDNA3.1. The effect of ectopic CHD5 expression on the growth of gastric cancer cells AGS was determined with monolayer colony formation assay. The forced expression of CHD5 in AGS cells was confirmed by RT-PCR (Fig. 4A). The number of colonies formed on the plate by cells over-expressing CHD5 was significantly reduced (*p *< 0.01) (Fig. 4B and 4C), indicating that CHD5 can suppress the growth of gastric cancer cells.

## Discussion

Over the past several years, many TSGs have been found to be epigenetically inactivated in gastric cancer, indicating that epigenetic silencing of TSGs is one of major molecular alterations in the process of gastric carcinogenesis [22-25]. In this study, CHD5 was identified as another potential TSG whose epigenetic inactivation may contribute to gastric carcinogenesis. It was frequently down-regulated through promoter hypermethylation in gastric cancer cell lines. The ectopic expression of CHD5 led to the growth inhibition of gastric cancer cells, indicating that CHD5 functions as a TSG epigenetically silenced in gastric cancer.

Promoter hypermethylation of CHD5 in cancer has been observed in other cancers [17,20]. However, the incidence of CHD5 promoter methylation in gastric cancer cell lines and tumors was found in this study to be relatively high, compared to the frequency of CHD5 methylation in other cancers (generally below 20%) [17,20]. Of course, more samples should be used to confirm this result with the same MSP primers in the following studies. Nevertheless, our finding suggests that CHD5 inactivation might be mediated by different mechanisms in different tissues. Whereas CHD5 is inactivated through copy number abnormality in various cancers [15,16], comparative genomic hybridization (CGH) indicated that 1p36, the CHD5-containing gene locus, is not significantly imbalanced in gastric cancers [26]. Instead, as shown in this study, CHD5 appears to be silenced predominately by promoter hypermethylation in gastric cancer.

There is growing evidence that hypermethylation of TSG promoter represents one of major molecular alterations in cancer development. The high incidence of CHD5 promoter hypermethylation in gastric cancer can be explored not only as a gastric cancer diagnosis but also prognosis prediction. To this end, it is important to characterize the CHD5 promoter methylation in association with clinical characteristics, such as age, gender, H. pylori infection, tumor grade, Lauren classification and differentiation. Given that the high heterogeneity of primary gastric carcinoma tissues, methylation analyses with higher resolution such as quantitative methylation specific analysis using sequenom or Taqman real-time PCR will be helpful to assess whether CHD5 promoter methylation is useful for early gastric cancer detection and prognosis prediction.

Although promoter methylation frequently inactivates CHD5 in gastric cancer cell lines, we cannot exclude the presence of other mechanisms for the loss function of CHD5 in gastric cancer. CHD5 expression is extremely low in MKN28 and SNU16 (Fig. 1), however, the promoter of CHD5 was only partially methylated in these two cell lines (Fig. 2B), indicating that other mechanisms may be responsible for the silencing of CHD5 in some of gastric cancer cell lines.

## Conclusion

CHD5 was frequently down-regulated through promoter hypermethylation in gastric cancer cells. The ectopic expression of CHD5 led to growth inhibition of gastric cancer cells, indicating that CHD5 functions as a tumor suppressor gene epigenetically silenced in gastric cancer.

## Competing interests

The authors declare that they have no competing interests.

## Authors' contributions

XW and YWL designed the study and wrote the paper. XW, KKL and LKS performed the experiments and analyzed the data.
